# Current state of the art of regional hyperthermia treatment planning: a review

**DOI:** 10.1186/s13014-015-0503-8

**Published:** 2015-09-17

**Authors:** HP Kok, P. Wust, PR Stauffer, F Bardati, GC van Rhoon, J. Crezee

**Affiliations:** Department of Radiation Oncology, Academic Medical Center, University of Amsterdam, Meibergdreef 9, 1105 AZ Amsterdam, The Netherlands; Department of Radiation Oncology, Charité Universitätsmedizin Berlin, Berlin, Germany; Department of Radiation Oncology, Thomas Jefferson University, Philadelphia, PA USA; Department of Civil Engineering and Computer Science, University of Rome Tor Vergata, Rome, Italy; Department of Radiation Oncology, Erasmus MC Cancer Institute, Rotterdam, The Netherlands

## Abstract

Locoregional hyperthermia, i.e. increasing the tumor temperature to 40–45 °C using an external heating device, is a very effective radio and chemosensitizer, which significantly improves clinical outcome. There is a clear thermal dose-effect relation, but the pursued optimal thermal dose of 43 °C for 1 h can often not be realized due to treatment limiting hot spots in normal tissue. Modern heating devices have a large number of independent antennas, which provides flexible power steering to optimize tumor heating and minimize hot spots, but manual selection of optimal settings is difficult. Treatment planning is a very valuable tool to improve locoregional heating. This paper reviews the developments in treatment planning software for tissue segmentation, electromagnetic field calculations, thermal modeling and optimization techniques. Over the last decade, simulation tools have become more advanced. On-line use has become possible by implementing algorithms on the graphical processing unit, which allows real-time computations. The number of applications using treatment planning is increasing rapidly and moving on from retrospective analyses towards assisting prospective clinical treatment strategies. Some clinically relevant applications will be discussed.

## Background

Hyperthermia, i.e. increasing the tumor temperature to 40–45 °C, is a very effective radiation and chemosensitizer. More than 20 randomized clinical trials have demonstrated the significant improvement in clinical outcome from adding hyperthermia to standard treatment regimens of radiation and/or chemotherapy [1]. Examples of tumor sites for which adding hyperthermia proved effective are cervical cancer [2], malignant melanoma [3], recurrent breast cancer [4], soft tissue sarcoma [5] and bladder cancer [6]. Deep-seated tumors, for example in the pelvic region, are usually heated with locoregional hyperthermia.

Modern locoregional heating devices usually consist of a large number of radiofrequency antennas organized in multiple rings [7–9]. The antennas surround the patient, so heating of normal tissue is inevitable with this technique. Although clinical results are good, the pursued optimal thermal dose of 43 °C for 1 hour is often not achieved due to treatment limiting hot spots in normal tissue, which impede further increase of total power. Since there is a clear thermal dose-effect relation [10–12], clinical outcome could improve further if power limiting hot spots are prevented. This makes high quality hyperthermia of utmost importance [13].

Active treatment control is essential to reduce the influence of hot spots and is highly dependent on reliable temperature information during hyperthermia as well as a good spatial power control to optimize the temperature distribution. Temperatures are usually measured by a small number of (minimally) invasive thermometry probes, and the sparse irregular sampling of temperatures does not provide adequate characterization of the 3D temperature distribution. Non-invasive thermometry (NIT) by e.g. MRI [14–18] is very useful to obtain more insight into the quality of heating, but is not generally available and restricted to a limited number of tumor sites; e.g. NIT is presently not feasible for moving tumors (abdomen) or for heterogeneous tissues. In a recent review Fani et al. described the growing interest in NIT using CT and conclude that the improvement of CT technology and the ability to measure in tissues (e.g. fat) where MR-thermometry is prone to inaccuracy make its exploitation attractive, though additional research is required for a final conclusion [19]. Using NIT based on MRI or CT during hyperthermia may require extensive and costly adaptations to the heating equipment when this consists of metal structures.

Spatial power control depends on the number of antennas and the operating frequency: the larger the number of antennas and the higher the frequency, the better the steering possibilities. A higher frequency provides a smaller focus volume, but is associated with a lower penetration depth and hence a larger number of antennas is needed for adequate heating of the of deep-seated tumors. Moreover, the large number of degrees of freedom, i.e. the amplitudes and phases of the individual antennas, make it very difficult for the operator to determine the optimal steering strategy by intuition or trial and error.

Therefore, hyperthermia treatment planning (HTP) is an essential instrument in modern locoregional radiofrequency hyperthermia treatments. With treatment planning, power absorption and/or temperature patterns in the patient are simulated to help the operator visualize the effect of different steering strategies. In addition, numerical optimization techniques predict phase-amplitude settings for optimal tumor heating, while accounting for user-defined constraints on temperatures or power absorption levels in normal tissue.

Significant progress has been made in the development and application of simulation techniques for HTP [20]. While for many years HTP has been used primarily as a research tool for retrospective verification of clinical treatment capabilities [21–23], HTP is nowadays used more often before and during clinical treatments to improve therapy [24–27]. For example, HTP is used to assist applicator configuration and system design, to compare the heating effectiveness of different devices for individual patients and to assess the impact of metal implants [28–35]. Furthermore, recent applications include assistance in phase-amplitude steering during treatment [16,24,25,36]. The clinical relevance of HTP is emphasized in the recent Quality Assurance Guidelines for clinical application of locoregional hyperthermia [37,38]. These guidelines mention HTP as a useful part of the hyperthermia treatment and requirements for HTP are specified.

In the regular workflow of treatment planning for regional radiofrequency hyperthermia, the following main steps can be distinguished:

i. Tissue segmentation for dielectric and thermal model generation
ii. Electromagnetic field simulation
iii. Temperature calculation
iv. Phase-amplitude optimization

For each step, dedicated simulation techniques are required and a wide variety of distinct methods are available. Methods may differ in accuracy, modeling complexity, clinical usefulness in terms of simulation time and/or pre-processing time, etc.

This review paper presents a state of the art overview of electromagnetic and thermal simulation techniques for locoregional HTP. First, segmentation procedures for dielectric model generation are described, followed by electromagnetic field simulation methods. Next, several thermal modeling techniques with varying complexity are discussed and an overview of phase-amplitude optimization methods is presented. Following some examples of clinically relevant HTP applications, an overview of available software packages for HTP will be given and future perspectives discussed.

## Hyperthermia treatment planning

### Dielectric model generation: tissue segmentation

Tissue segmentation is a very important aspect of HTP. Dielectric properties, which determine the energy absorption in tissue, vary significantly between different tissues and organs in the human body [39]. Thus, tissue segmentation strongly influences the simulation results. Segmentation is based on a CT or MRI scan in the same position as during hyperthermia treatment and can be performed manually or (semi-)automatically. Advantages of MRI data over CT are the very good soft tissue contrast and the absence of an additional radiation dose to healthy tissues. The tumor or target region should be outlined manually to allow comparison of different treatment plans in terms of target coverage and thus treatment quality.

Automatic segmentation based on CT Hounsfield Units has been described by Hornsleth et al. [40]. Using this method basic tissue types with a large dielectric contrast can be distinguished (i.e. muscle, fat, bone and lung/air). A drawback of this technique is that organs that are known to have the same density but very different dielectric and/or thermal properties are segmented as regular muscle tissue (e.g. liver and kidneys). This can be overcome with additional manual delineation of relevant organs to improve the accuracy of the model.

Wust et al. have shown that absorbed power calculations based on manually delineated dielectric models yield a much better correlation with measurements during treatment than calculations using models created by Hounsfield Unit based automatic segmentation techniques [41]. Requirements to normal tissue delineation for HTP are higher than for radiotherapy treatment planning, because of the large variation in dielectric and thermal properties between different organs and tissue regions, and in particular the electromagnetic boundary conditions (4th Maxwell equation). A drawback of manual delineation for HTP is the required operator time, but similar problems have been solved for radiotherapy planning systems, and contouring modules are becoming increasingly potent and user-friendly.

For HTP multiatlas-based delineation of head and neck CT images has been developed [42]. This semi-automatic delineation method requires a library of accurately segmented patient models, in which organs show all relevant tissue-shape variations. To improve accuracy of the segmentation, the multiatlas registration was combined with an intensity model to distinguish between foreground and background [42]. Results were compared to manual delineations by three trained observers and for the majority of tissues the segmentation accuracy was close to the interobserver agreement.

Verhaart et al. investigated the possible benefit of using combined (manual) CT-MRI based segmentation for head and neck hyperthermia [43]. They showed that MRI allowed a more detailed delineation of the brain, which had a minor impact on the simulated absorbed power, but it allows more accurate temperature predictions. Therefore, combined CT-MRI atlas-based delineation for head and neck hyperthermia is currently under development [43]. Atlas-based segmentation for HTP in the pelvic region has not been investigated yet.

After segmentation using either of the above mentioned methods dielectric tissue properties from literature are assigned, which show a large spread (~50 %) [39]. This uncertainty in dielectric tissue properties yields an inaccuracy of ~20 % in both specific absorption rate (SAR) and temperature predictions [44]. This demonstrates the need for patient-specific dielectric properties to achieve accurate treatment planning.

To realize patient-specific HTP, dielectric imaging techniques that reconstruct a full 3D distribution of the patient’s dielectric tissue properties are investigated. Farace *et al.* proposed a two-step dielectric imaging method: first the water and fat content are determined using standard MRI techniques, then the water contents are converted to permittivity maps [45]. This method was tested on a human volunteer and dielectric properties at 120 MHz and 1 GHz were reconstructed for CSF, liver, kidney, fat, spleen, muscle, cancellous bone, bone marrow, vitreous humour, white and grey matter [46]. The variation within each tissue type was typically < ~10 %. However for some tissues (e.g. the liver) deviations with respect to measured values were more than 20 % [46] and therefore the method requires further improvement for standard use in HTP.

Recently, the use of Electric Property Tomography (EPT) for quantitative reconstruction of dielectric properties has been investigated [47,48]. EPT is an MRI-technique that derives dielectric properties from the measured transmit *B*_1_^+^ amplitude and phase maps. Reconstructed conductivity values in a phantom tumor model corresponded within 10 % with probe measurements [47]. Next, the B_1_^+^ distribution in a dielectric human model was simulated and the result was used to mimic a measurement and test the feasibility to reconstruct conductivity maps in the human pelvis [47]. A very good resemblance was found, for example, the reconstructed tumor conductivity was 0.86 S m^−1^, where the input value was 0.90 S m^−1^. This method works particularly well in the central area of the pelvis and homogeneous regions. The EPT reconstruction algorithm assumes locally (piece-wise) constant dielectric properties, but is not accurate at the boundaries between those regions, i.e. at tissue interfaces.

Since tissue interfaces are quite numerous, this problem needs to be resolved for EPT to be valuable for accurate HTP. For this purpose Contrast Source Inversion-Electric Property Tomography (CSI-EPT) is currently under development [49]. This method considers EPT as a full electromagnetic inversion problem and the Contrast Source Inversion method [50] is applied to B_1_^+^ data to obtain dielectric properties. This is an iterative method, minimizing an objective function that measures the discrepancy between measured and modeled data. Balidemaj et al. demonstrated the feasibility of this method in 2D by showing reliable reconstructed maps of dielectric properties including tissue interfaces [49]. Future work involves extension of the method to 3D and inclusion of techniques to suppress noise [49].

### Electromagnetic field calculation techniques

After the dielectric patient model has been created, the applicator model is combined with the patient model and the electromagnetic (EM) field distribution in the patient is calculated by numerically solving Maxwell’s equations. For accurate clinically representative simulations, the patient’s position in the heating system used for HTP should correspond to the positioning used during clinical treatment. For an operating frequency around 70 MHz the deviation in positioning should be smaller than 1–2 cm [51,52]. At higher frequencies more accurate positioning might be necessary, since a higher frequency is associated with a smaller wavelength of the EM field and thus a smaller hot spot size [53–55]. Several EM-simulation methods have been described in the literature [56,57], either based on the differential or integral form of Maxwell’s equations. To avoid reflections of the electromagnetic waves at the boundaries of the computational domain, absorbing boundary conditions are essential. Several variations exist [58–61], of which the perfectly matched layer is the most effective [59].

#### Differential techniques

The finite difference time domain method (FDTD) requires discretization of the computational domain into rectangular voxels [62]. For accurate EM-simulations at least 10–20 voxels per wavelength should be used. The standard FDTD method is based on the Yee cell, in which the electric field components are located at the edges of the voxels and the magnetic field components are located at the faces, or vice versa [63]. The propagation of the electromagnetic field is calculated for successive time steps, until steady state is reached. The FDTD method is a very popular method because of its efficiency in terms of memory use and computation time, but a drawback is the occurrence of so-called stair-casing at highly irregular and geometrically detailed tissue interfaces. The use of very high resolution, a non-uniform grid or post-processing interpolation techniques (in case of contour-based segmentation) [64] can reduce these artefacts, although at the cost of increased complexity and computation time.

The finite element (FE) method is also based on differential equations [65,66]. The FE method has the advantage of better accuracy at irregular tissue interfaces, compared to FDTD. However, grid generation is more complicated and dedicated mesh generation software is required to subdivide the computational domain into tetrahedral (or hexahedral) elements [67,68]. Usually small mesh elements are used in highly detailed heterogeneous regions and larger mesh elements in relatively homogeneous regions. The FE method uses trial functions; i.e. a set of basis functions defined over the mesh elements that comprise the entire problem domain, including the boundary conditions. The approximation error is minimized by fitting these trial functions to the partial differential equations. Thus, the field equations are represented in terms of polynomials with unknown coefficients defined on the mesh nodes or along element edges. These unknown coefficients are determined by solving a sparse matrix equation.

#### Integral equation methods

The finite integration technique (FIT) [69] can be considered as a generalization of the FDTD method and also shows some resemblance to the FE method, as it can be used with rectangular or tetrahedral meshes. The integral equations are transformed into a set of matrix equations on an orthogonal dual grid pair. The electric grid voltage and magnetic facet flux are defined on the primary grid, whilst magnetic grid voltages and electric facet fluxes are defined on the second grid. The transient solver is based on the solution of these matrix equations, which yields a fully explicit time-stepping procedure as in the FDTD method. The FIT is very flexible in geometric modeling and handling of boundaries.

The volume surface integral equation (VSIE) method has been proposed by Wust et al. for use in combination with a region-based segmented patient model, discretized on a tetrahedral grid [70]. The advantage of this method is that it is very accurate at tissue interfaces of any shape or geometry, provided that the interfaces are described accurately by the grid. This high accuracy is realized by splitting the integral equation into a volume integral and a surface integral at the interface. The volume surface integral equation is solved by the iterative method GMRES. In terms of computation time, however, the VSIE method is less efficient than the FIT. Furthermore, because of the tetrahedral grid required, dedicated mesh generation software is needed.

The weak form of the conjugate gradient fast fourier transform method (WF-CGFFT) has been described by Zwamborn et al. [71,72]. In this method the domain-integral equation is weakened by a testing and expansion procedure. The resulting weak form of the integral equation is then used as input for the CGFFT method, which solves a set of coupled convolution integral equations by combining a conjugate gradient iterative solver (CG) and a fast fourier transform (FFT). A drawback of this method is the relatively slow convergence of CG. Furthermore, the use of a rectangular grid again yields the possible occurrence of stair-casing at tissue interfaces.

### Thermal modeling

Thermal modeling is important to provide insight in the 3D temperature distribution, which is determined by the SAR deposition and cooling mechanisms as thermal conduction, perfusion and bolus cooling. Perfusion is the most important cooling mechanism, which is dependent on both temperature and time and can increase up to a factor of 10 during hyperthermia [73]. However, clinical data of a large number of patients, tumor entities and heat sessions suggested less variability during locoregional hyperthermia [74,75]. Because of the significant heat removal by blood flow, thermal modeling is a very important and challenging aspect of HTP. At present, the models use predicted values of perfusion based on the literature, which implies some uncertainties in temperature predictions [76]. Three-dimensional perfusion measurements are difficult to perform during hyperthermia, and therefore thermal simulations based on literature values are still common practice. Despite these uncertainties, thermal modeling is essential to account for cooling mechanisms in treatment planning.

A wide variety of thermal models has been developed, with varying complexity [77,78]. These models can be subdivided into continuum models, i.e. models in which perfusion is described by a continuum flow model, and discrete vasculature models, i.e. models in which thermally significant blood vessels are modeled discretely to predict local temperature inhomogeneities.

#### Continuum models

The most commonly used method is the bio heat transfer equation, proposed by Pennes in 1948 [79]. This model describes the impact of perfusion by an isotropic heat sink, proportional to a non-directional volumetric average perfusion rate and local temperature rise. The model assumes that the equilibration length, i.e. the axial distance it takes before the blood is thermally equilibrated with the surrounding tissue, is infinite everywhere and zero in the capillaries. Therefore, this model does not account for heat exchange between tissue and realistic vasculature. Another disadvantage is that the direction of blood flow is not taken into account.

Several adaptations to this model have been proposed to account more accurately for blood flow. Wulf replaced the non-directional perfusion term in the Pennes model by a uni-directed flow [80], which accounts for interaction between blood and tissue. However, this non-directional perfusion does not provide an adequate description of counter-current flow. In this case the net flow is zero and the model erroneously predicts that the blood flow does not contribute to heat transport.

The enhanced effective conductivity model is another extension of the Pennes model to account for directional blood flow [81]. This model describes convection due to blood perfusion by an effective conductivity term, represented by a tensor. A more generalised version of this model has been described by Weinbaum and Jiji [82]. The effective conductivity approach is well-suited to model the impact of relatively smaller vessels (i.e. <~0.5 mm diameter). The presence of large thermally significant vessels should be accounted for by discrete vasculature models.

#### Discrete vasculature models

Basic discrete vessel models demonstrated that heat transfer between large thermally significant blood vessels and tissue is significant and should be accounted for in hyperthermia treatment planning [83–87]. Significant progress has been made in thermal modeling with discrete vessel networks [78] and advanced 3D models with branching vessel networks have been developed, with varying levels of complexity and flexibility for clinical use [88–92].

Discrete vessel models consisting of straight vessel segments provide limited flexibility to model realistic detailed and curved vasculature, because the maximum diameter for accurate modeling of vessels is closely related to the voxel dimensions of the tissue grid [88,92]. Therefore, Mooibroek and Lagendijk developed a more realistic model with a semi-curved representation of vessel networks [89]. This model labels nodes with their centres closest to the vessel axis as so-called ‘vessel nodes’, thereby subdividing the model geometry into a vessel space and a tissue space. In contrast to the preceding model by Lagendijk [88], branching, bending, obliqueness, widening and tapering of vessels are preserved. Despite these substantial improvements, the model is impractical for clinical use, because a very small grid spacing is required for accurate description of true 3D vessel networks.

To overcome these problems Kotte *et al.* developed a sophisticated discrete vasculature model (DIVA) that allows a true representation of 3D patient-specific vessel networks [90,93]. The model geometry is again subdivided into a vessel space and a tissue space, but in DIVA vasculature is described by 3D curves with a specified diameter, yielding a grid-independent vessel description. Furthermore, the 3D curve representation of blood vessels makes the model compatible with CT/MRI angiography vessel reconstruction software, which is important for routine use in treatment planning. The heat flow between vasculature and tissue is estimated using a semi-analytical approach. The model has been validated experimentally in isolated perfused animal tissue [94]. Comparison of measured and simulated temperature profiles showed very good agreement, with deviations on the order of the experimental error [94].

Applications of the DIVA model for locoregional HTP have been described for extremity sarcomas and pelvic heating [95,96]. Van den Berg et al. used the DIVA model to simulate locoregional hyperthermia of the pelvic region [96]. Quantitative 3D perfusion maps of the prostate and a vessel model of the pelvis were reconstructed using dynamic contrast-enhanced CT imaging. A detailed model of the prostate vasculature was reconstructed from a post-mortem prostate. The DIVA model was combined with the 3D perfusion map and simulation results were compared to Pennes’ bio heat model with either a homogeneous prostate perfusion or the 3D perfusion map. Modeling of the 3D heterogeneous prostate perfusion instead of a homogeneous perfusion was found to result in 1-2 °C lower tumour temperature [96]. Comparing DIVA with the Pennes model using 3D perfusion maps showed differences up to 0.5 °C [96]. Furthermore, Craciunescu et al. compared temperature simulations with different thermal models to measurements with MR thermometry for heating a patient with a high grade sarcoma using an annular phased array system [95]. Results achieved with DIVA using reconstructed vasculature from MR angiography combined with perfusion maps corresponded best to MR thermometry. This demonstrates that thermal modeling including discrete vasculature is essential for reliable HTP.

Thermal simulations with discrete vasculature allow more reliable temperature predictions. However, the DIVA model is rather computationally expensive as it calculates the evolving temperature distribution by applying many small time steps until a user-defined end time, which is usually steady-state. This yields calculation times up to several hours, which makes it impractical for routine clinical applications. To overcome this problem Kok et al. developed a very efficient method to calculate the steady-state temperature distribution. The method represents the bio heat transfer and the interaction between vasculature and tissue by a linear system, which is solved using an iterative method [91]. For further speed-up the method was implemented on the graphical processing unit (GPU), which makes it suitable for real-time simulations [91].

For clinical application discrete vessel input for the DIVA model will be reconstructed from CT or MR angiography, though not all thermally significant vessels might be visible. To add vasculature to incomplete vessel networks, or to create realistic artificial vasculature, vessel generation software has been developed [97,98]. Full networks consisting of arteries and veins can be constructed and the presence of bone and cavities can be taken into account.

### Treatment optimization techniques

Adequate phase-amplitude steering is essential to optimize tumor heating while avoiding treatment limiting hot spots. Optimization techniques can be very useful for this purpose, since finding the most effective phase-amplitude setting for modern heating devices consisting of a large number of antennas is by no means a trivial task. Various optimization strategies for phased array systems have been developed, which can be subdivided into SAR-based and temperature-based optimization methods.

#### SAR-based optimization

A very efficient way of optimization is by maximizing the quotient of the absorbed power in the tumor region and a weighted energy norm outside the tumor using an eigenvalue technique [99,100]. User-defined weight functions can be defined to account for specific regions where hot spots are expected, or regions which are known to have a significantly different perfusion, though in general these methods provide limited flexibility in formulation of the objective function to be optimized.

More flexibility is provided by genetic algorithms or variants such as particle swarm optimization [101,102], which make use of random methods to iteratively improve a candidate solution and determine an optimum. These methods are useful to approach global optima, but the precision to determine the global optimum is low. This can be overcome by combining a genetic algorithm with a local optimization strategy, such as a line search method [103].

The quality of the optimization result is strongly dependent on the objective or goal function selected to optimize. Canters *et al.* applied a variety of quality indicators that are often used for SAR characterization as a goal function during optimization to assess their suitability for optimization purposes [104]. The correlations between the SAR indicators and the target temperatures predicted using the Pennes’ bio heat equation were determined, which showed that the hot spot-target SAR ratio (i.e. the ratio between the average SAR in the first volume percentile and the volume averaged SAR in the target) is the most suitable objective function for SAR-based optimization.

SAR-based optimization can be very useful to improve tumor temperatures, as there is a correlation between SAR and temperature [105]. However, a general drawback of SAR-based optimization techniques is the difficulty to accurately account for relevant cooling mechanisms, such as perfusion, conduction and bolus cooling. Modeling of these mechanisms is essential for correct prediction of treatment limiting hot spots and to prescribe antenna settings to avoid them. A study by De Greef *et al.* showed that temperature-based optimization is preferable to SAR-based optimization [76].

#### Temperature-based optimization

Temperature-based optimization maximizes the tumor temperature with constraints to normal tissue temperatures. This can be done using either hard constraints or soft constraints. With soft constraints, the constrained optimization procedure is approximated by an unconstrained optimization by adding a penalty term to the objective function. This penalty usually summates the squares of the temperature exceeding from the constraints [106,107]. Constrained optimization is more susceptible to returning a local minimum instead of the global minimum. This can be avoided by performing multiple runs with multiple random initial phase-amplitude settings.

During the optimization process a substantial number of temperature calculations are performed. Calculation by solving the corresponding differential equation is extremely computationally intensive and therefore, efficient methods have been developed. Das et al. proposed a method to calculate the temperature distribution by superposition of pre-computed distributions [107]. In addition, some techniques for model reduction have been proposed to decrease computation time, e.g. by using tissue groups rather than individual points. In this case the tissue groups consist of points that achieve their maximum heating potential for approximately the same phase-amplitude setting [107]. Cheng et al. introduced a virtual source approach, which combines a selection of pre-calculated phase-amplitude settings (the so-called virtual sources) that are most likely to heat the tumor, thereby assuming that the optimal phase-amplitude setting can be represented as weighted combinations of these virtual sources [108]. This allows very efficient computations and thus real-time use.

**Fig. 1:**
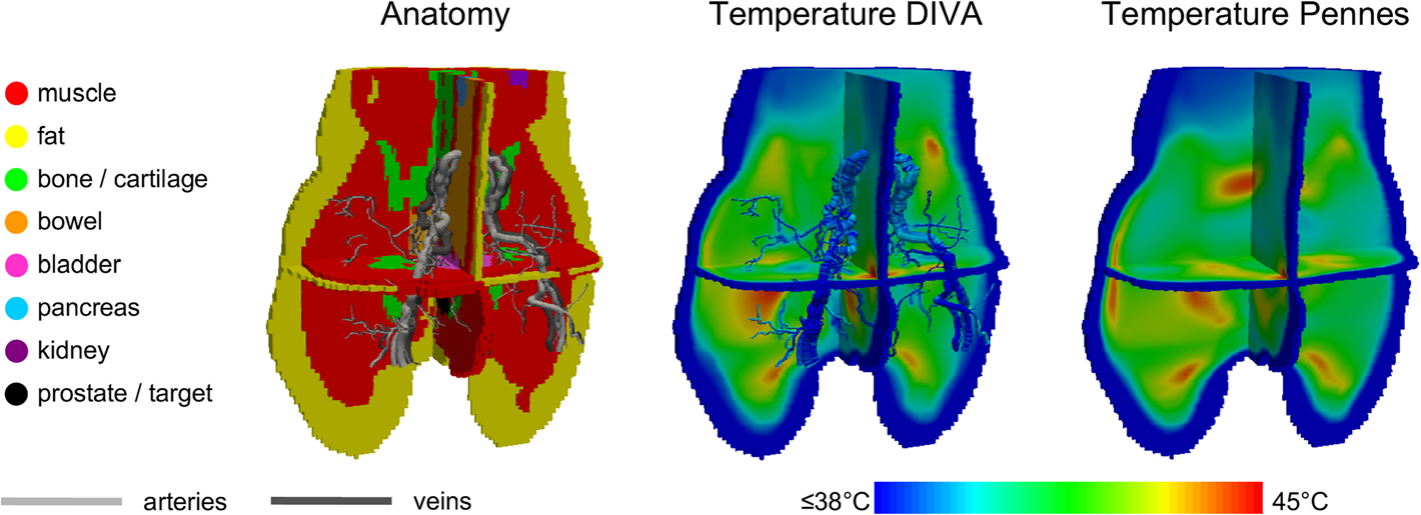
Phase-amplitude optimization based on DIVA. Modeled human anatomy with optimized temperature distributions for locoregional heating of the pelvic region with the prostate as target using the 70 MHz AMC-8 system. Optimization with (central picture) and without (right picture) taking discrete vasculature into account yield substantially different predicted optimal temperature distributions, demonstrating the value of incorporating discrete vasculature in optimization routines. This figure was reproduced from Kok HP, Van den Berg CAT, Bel A, Crezee J: Fast thermal simulations and temperature optimization for hyperthermia treatment planning, including realistic 3D vessel networks. MedPhys 2013, 40:103303. copyright © 2013, American Association of Physicists in Medicine. Reproduced with permission of the American Association of Physicists in Medicine

Temperature-based optimization methods are usually based on Pennes’ bio heat model, as this is computationally least expensive. Recently, Kok et al. developed an efficient optimization method based on DIVA [91]. The tissue and blood temperatures and the power were written as a vector–matrix–vector multiplication, which facilitated extension of the concept of superposition of pre-computed temperature distributions mentioned above to the DIVA model [91]. Figure 1 shows a comparison of optimization based on Pennes’ bio heat model and the DIVA thermal model.

## Applications of Hyperthermia Treatment Planning

**Fig. 2:**
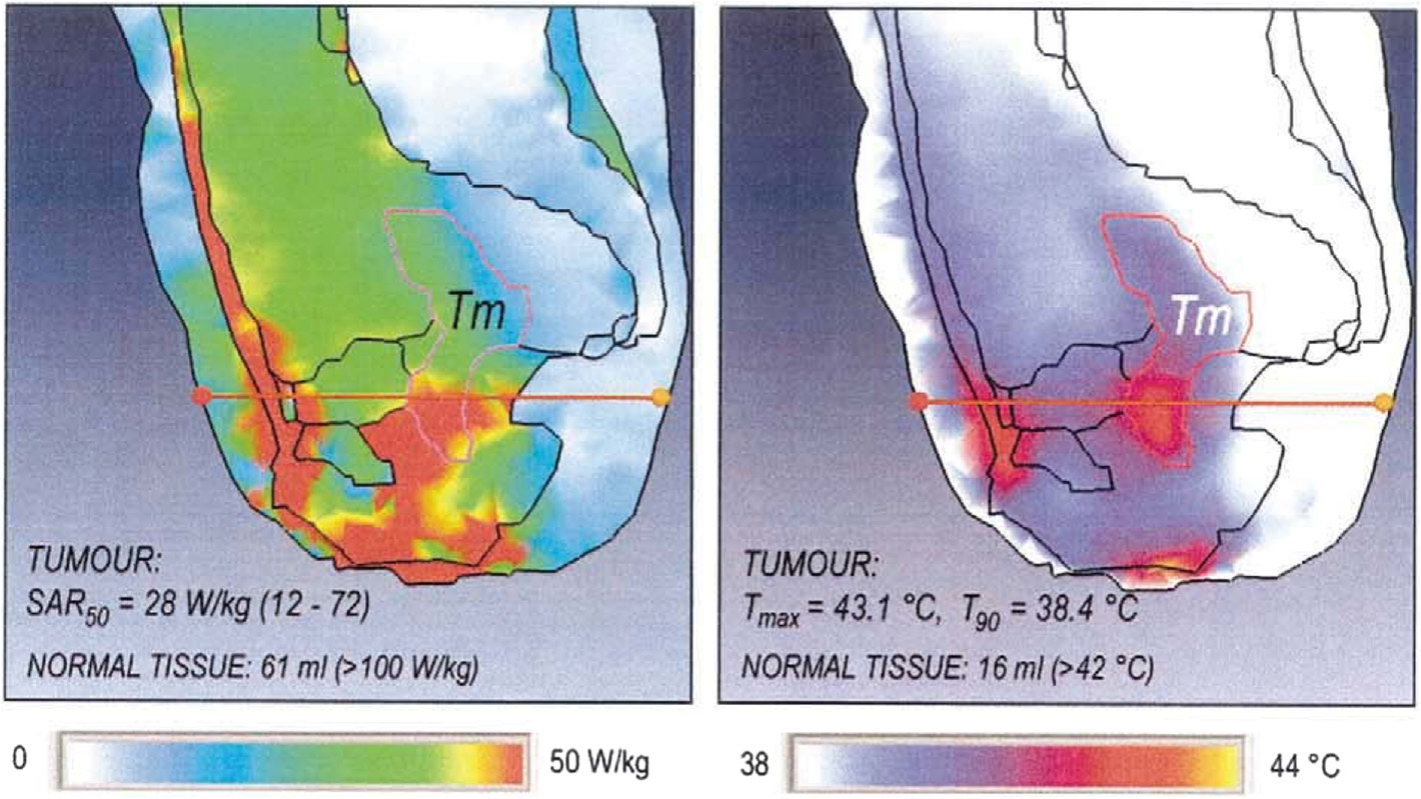
Hot spot prediction by HTP. Example of the calculated SAR (*left*) and temperature (*right*) distribution for a cervical carcinoma patient where clinical intolerances were observed during locoregional hyperthermia. The patient complained of discomfort in the vaginal and symphyseal regions, which was correctly predicted by the retrospective planning calculations. This picture was reproduced from Sreenivasa G, Gellermann J, Rau B, Nadobny J, Schlag P, Deuflhard P, Felix R, Wust P: Clinical use of the hyperthermia treatment planning system HyperPlan to predict effectiveness and toxicity. Int. J. Radiat. Oncol. Biol. Phys 2003, 55:407–419. copyright © 2003, Elsevier. Reproduced with permission of Elsevier

The number of applications using HTP is increasing rapidly. Until a few years ago applications were mainly of retrospective nature and aimed at verification for clinical use. Some research groups demonstrated a correlation between simulations and measurements during hyperthermia treatments [21–23]. Sreenivasa et al. showed that treatment planning can predict treatment limiting hot spot locations (Fig. 2) as well as distinguish between patients who should be easy to heat or difficult to heat [22].

**Fig. 3:**
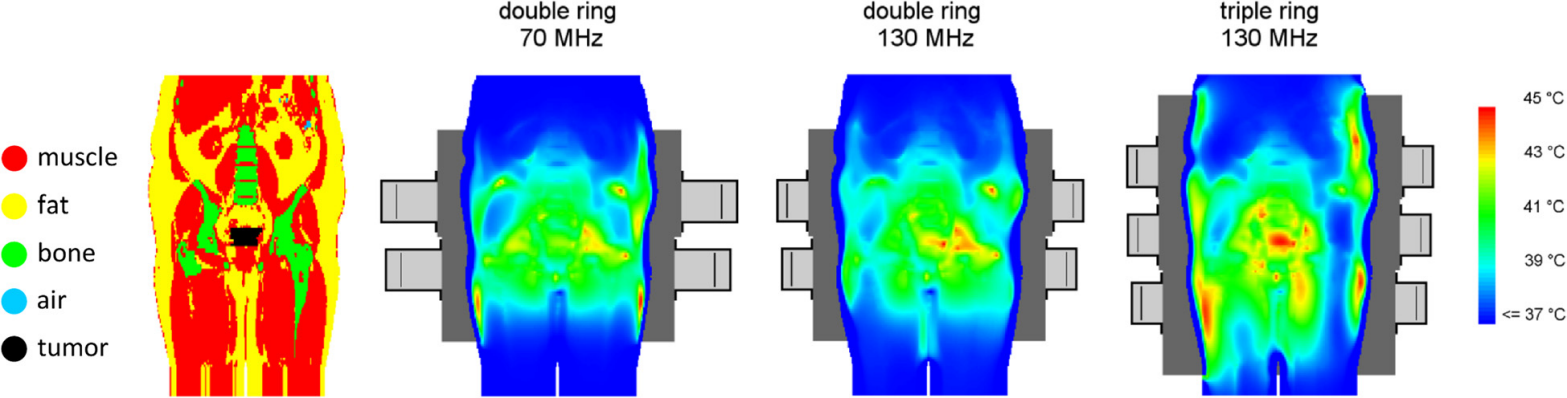
- Analysis of heating effectiveness by HTP. Treatment planning example demonstrating the influence of operating frequency and the number of antennas and rings on the heating effectiveness of a cervical cancer patient. The picture on the left shows the patient anatomy and the other pictures show the simulated temperature distributions for heating with a double ring system at 70 MHz or 130 MHz and a 130 MHz triple ring system. Both higher frequency and increasing number of antennas improve the steering properties and heating effectiveness. The simulated T_90_ was 40.3, 40.9 and 41.9 °C for the three cases, respectively. This figure was reproduced from Kok HP, De Greef M, Borsboom PP, Bel A, Crezee J: Improved power steering with double and triple ring waveguide systems: the impact of the operating frequency. Int. J. Hyperthermia 2011, 27:224–239, copyright © 2011, Informa Healthcare. Reproduced with permission of Informa Healthcare

Another important application of treatment planning is system design and comparison of heating patterns from different devices as a means of determining the best steering configuration to avoid hot spots and optimize tumor heating. Kroeze *et al.* used treatment planning to demonstrate that radiative heating systems are generally preferable to capacitive systems for adequate heating of pelvic tumors, due to the risk of overheating high resistivity superficial fat by capacitively coupled electric currents [28]. Furthermore, several studies have been performed on the influence of operating frequency and the number of antennas and rings [29-33]. An increasing number of antennas improve the steering properties and combined with a properly selected operating frequency this is expected to improve tumor temperatures (Fig. 3). However, considering that complexity increases rapidly with the number of antennas, a compromise between heating efficacy and system robustness and control is necessary to keep the system manageable in clinical practice [33].

In recent years, a stronger focus is emerging on the use of treatment planning to assist the treatment strategy. A study applying prospective treatment planning with temperature-based optimization showed that treatment planning can improve heating efficiency [109]. Using phase-amplitude settings prescribed by treatment planning, a lower amount of power was needed to obtain the same tumor temperature rise compared to phase-amplitude settings determined by standard clinical practice [109].

Prospective treatment planning is challenging because of the uncertainties in tissue properties and a priori unknown changes in perfusion levels during hyperthermia. This is particularly true for complex three-dimensional multi-antenna applicators such as the SIGMA-Eye applicator (three rings with 12 antenna pairs) [110]. These difficulties increase with frequency. A simulation study suggested that phase-amplitude optimization would result in a temperature gain > 1 °C for the three-dimensional SIGMA-Eye applicator in comparison to the two-dimensional SIGMA-60 applicator [106]. However, this theoretical advantage has not yet materialized in clinical practice. This could be explained by various errors, including uncertainties in tissue properties [33], phase errors, coupling effects and positioning errors, that accumulate and cause a significant difference between the predicted and actual optimum, particularly for complex multi-antenna systems. Seebass et al. demonstrated a rapid loss of the temperature gain in case of moderate variations in phases and/or positioning [30].

**Fig. 4:**
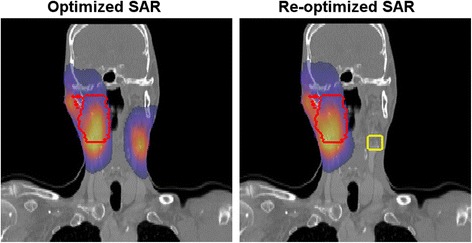
Hot spot reduction by re-optimization. Clinical example of successful re-optimization of the SAR distribution during head and neck hyperthermia [24]. The target region is indicated by the red contour. After the first optimization a hot spot at the other side occurred (left picture), which is successfully suppressed after re-optimization (right picture)

As discussed above, a pre-treatment plan might be suboptimal and power limiting hot spots might still occur when a pre-treatment plan is applied. When using MR guided hyperthermia, the calculated electric fields of the pre-treatment plan can be corrected using on-line measurements [15,111]. This improves the predictability and quality of the SAR distribution. Alternatively, the uncorrected electric fields can be used in adaptive planning to determine alternative antenna settings that suppress hot spots whenever they occur during treatment. Kok et al. demonstrated the feasibility of this on-line adaptive planning technique by showing that measured SAR changes after adapting antenna settings correlate to simulated SAR changes [26]. This strategy does not require the extensive on-line measurements provided in an MR guided heating system. First studies using HTP-guided steering for pelvic hyperthermia using the Sigma-60 system with adaptive SAR optimization showed rather equal performance, i.e. similar intraluminal temperatures compared to phase-amplitude steering by experienced operators [25]. Suppression of hot spots is expected to become more successful by using adaptive planning with more sophisticated optimization routines. Moreover, the Sigma-60 system has four antenna pairs and added value of HTP might increase with an increasing number of independent antennas. HTP-guided steering can also be very useful to adequately heat tumor sites for which limited clinical experience is available. As an example of this, Rijnen et al. described adaptive HTP with SAR optimization for two patients with head and neck tumors [24]. An approximate doubling of the target SAR as a result of HTP was demonstrated for one patient [24]. Figure 4 shows a clinical example of reducing a hot spot after re-optimization during head and neck hyperthermia.

The above mentioned studies demonstrate that on-line adaptive HTP is very promising to improve treatment quality using modern heating devices with a large number of antennas. Probably, the combination of MR thermometry feedback with on-line adaptive HTP can realize optimal patterns and focus heat into the target [16,36]. This approach provides an online correction for all deviations from the plan and can assist optimization of heating for each patient.

## Software packages for Hyperthermia Treatment Planning

**Fig. 5:**
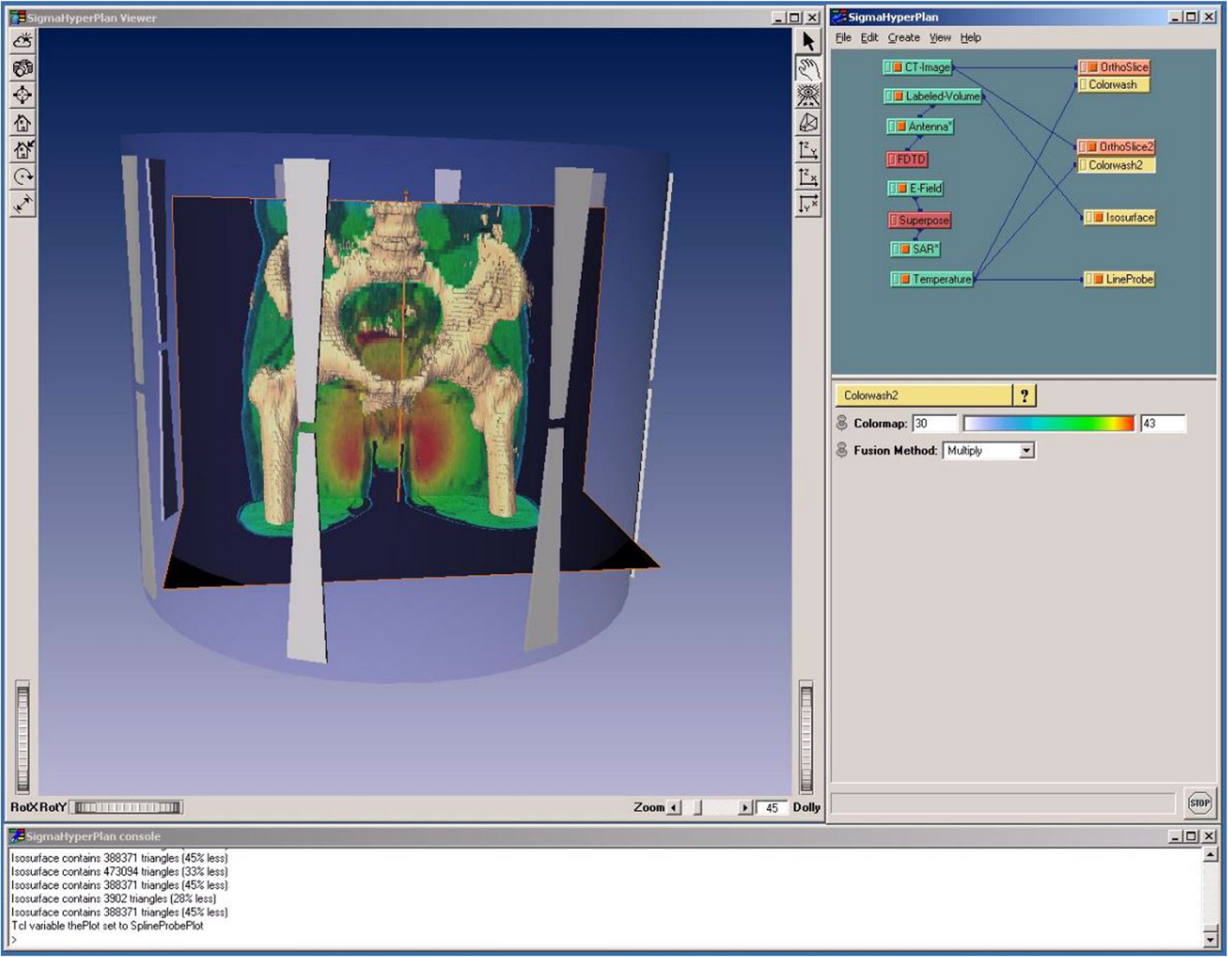
User interface of HyperPlan. Platform of the HyperPlan treatment planning system. The 3D viewer (left) is configured in the object pool (right above) by combining data sets (*green*) and procedures (*red*)

Since applications for HTP are increasing rapidly, the number of dedicated treatment planning software packages are also expanding. The most widely used commercially available treatment planning system for locoregional hyperthermia is Sigma HyperPlan (Fig. 5). This treatment planning system has been developed at the Konrad Zuse Institute and applies the FE-method with tetrahedral grids for E-field and temperature calculations [57]. Modules using the FDTD-method (contour-based as well as voxel-based) are also available [64]. Applicator models are at present only implemented for the range of SIGMA applicators (Dr Sennewald Medizintechnik, Munich, Germany).

Besides this integrated planning system more flexible treatment planning systems have been developed such as the AMC DIVA hyperthermia treatment planning system (AMC, Amsterdam, The Netherlands) and SEMCAD X (SPAEG, Zurich, Switzerland), of which the latter is commercially available. These planning systems are flexible and allow for SAR calculations, thermal modeling and phase-amplitude optimization based on SAR or temperature for various applicator systems.

Furthermore, electromagnetic calculations and basic thermal simulations for HTP can be performed using general purpose commercial software packages such as COMSOL (www.comsol.com), Ansys High Frequency Structural Simulator (HFSS; www.ansys.com) and CST STUDIO SUITE (www.cst.com). These packages also provide flexibility in applicator modeling, but additional software is required for tissue segmentation, more advanced thermal modeling and treatment optimization.

Commercial software is continuously improving and adapting to clinical needs. However, it should be noted that for some specific and/or relatively new applications which are not (yet) available in commercial treatment planning software packages, for example adaptive HTP, additional software tools will be needed and these should initially be developed in-house.

## Future perspective

There has been significant progress in the development of treatment planning software for hyperthermia and its integration into the clinical workflow is becoming common practice. Because of uncertainties in dielectric and thermal tissue properties and various other inaccuracies, pre-treatment planning results often do not represent the true optimal treatment plan. Real-time re-optimization has become possible by implementing algorithms on the GPU, which allows on-line adaptive HTP. This proves a good strategy to adapt the treatment plan in response to hot spots and improve the heating quality [24,26,36]. Therefore, the use of on-line adaptive HTP is expected to increase in the near future.

At the same time, research is in progress to reduce the impact of uncertainties in tissue properties in order to further improve the simulation accuracy of pre-treatment planning. MRI-based reconstruction of dielectric tissue properties using CSI-EPT is currently under development and results so far are very promising [47]. Further development of this method would allow reconstruction of patient-specific 3D maps of dielectric tissue properties for use in HTP. This will improve reliability of SAR calculations and pre-treatment SAR-based optimization. Moreover, CSI-EPT can also reconstruct the unknown electric field distribution in the patient and is therefore a promising method to determine the patient-specific SAR deposition for a hybrid heating device, provided that the RF heating frequency is equal to the spin excitation frequency (e.g. 64 MHz at 1.5 T) [53]. Reliable SAR calculations should provide a good starting point for on-line adaptive temperature-based optimizations that improve heating quality, since SAR and temperature are correlated. Regardless, additional on-line corrections of phase errors and positioning deviations provide further improvement in heating and can be ensured by on-line NIT using hybrid MR guided hyperthermia systems [17,112] or integrated systems [53]. This will improve the correlation between treatment planning and thermal dose measurements.

Perfusion imaging provides important input for thermal modeling [96,113], but accurate characterization of three-dimensional perfusion during hyperthermia is very difficult, which makes reduction of the impact of perfusion uncertainty very challenging. The uncertainty of blood perfusion varies as a complex function of temperature and time. Even so, better characterization of tissue properties for individual patients has been shown to improve the reliability of thermal simulations for clinical applications [114]. This strategy improves the determination of perfusion and thermal conductivity values for different tissues by minimizing the cumulative error between measured and simulated steady-state temperatures during treatment. Though this improves simulation accuracy, an uncertainty of about 2 °C remains [114]. The use of MR guided heating systems will facilitate measurement of tissue perfusion during treatment in addition to non-invasive thermometry, which will allow the user to evaluate and validate the thermal modeling and improve HTP with on-line adaptation.

Another promising approach to improve the reliability of HTP is the use of robust optimization techniques that consider model parameters as stochastic variables [115, 116]. With a stochastic approach, optimization would be based on expected temperatures and the expected value of T90 (i.e. the temperature exceeded in 90 % of the target region) would be a logical choice for goal function, since T90 is strongly correlated to clinical outcome.

Improving the accuracy of pre-treatment HTP is also important for reliable reconstruction of the delivered thermal dose during treatment. This will improve insight in the thermal dose-effect relation, which is usually based on a limited number of (invasive) temperature measurement locations. Furthermore, detailed knowledge about the actual temperature distribution enables adaptation of the treatment strategy based on accurate prediction of the effect of alternative treatment settings. This should further improve minimum temperature levels that will translate into improved tumor control.

The use of more advanced thermal modeling including discrete vasculature has been shown to improve temperature predictions and is expected to become more common in the near future, especially since the main bottleneck of computation times up to several hours has been overcome by the use GPU-based algorithms [91]. However, reconstruction of patient-specific vasculature as input for the DIVA thermal model still requires substantial manual interaction. Further developments will focus on minimizing this manual interaction for routine clinical application. Thus, for practical reasons the bio heat transfer equation is still frequently used for HTP, but further research will reveal the benefit of sophisticated thermal modeling for online optimization using NIT and adaptive HTP.

Future research is also expected to include combined treatment planning for radiotherapy and hyperthermia. The effect of radiosensitization by hyperthermia can be quantified in terms of equivalent dose distributions [117]. Recently, a theoretical framework and software tools were developed to calculate equivalent dose distributions, which allows bi-modality treatment planning once reliable linear-quadratic parameters are available for hyperthermic conditions [117,118]. This is the subject of on-going research. Bi-modality treatment planning makes it possible to optimize the additive effect of radiotherapy and hyperthermia treatments, rather than prescribe doses separately for the two modalities. This might eventually yield a patient-tailored treatment plan with the highest possible tumor control probability and minimal risk of normal tissue complications.

## Conclusions

There has been significant progress in the development and application of hyperthermia treatment planning software. While earlier applications were mainly for retrospective analysis, nowadays a stronger focus is emerging towards real clinical applications such as assisting phase-amplitude steering during treatment. This has become possible by advanced and computationally efficient algorithms that allow real-time calculations. In the coming years, the role of treatment planning during clinical hyperthermia (with or without MR monitoring) is expected to increase further, resulting in significant improvements in treatment quality.
